# Recombinant Artemin‐Fc Fusion Protein Attenuates TLR4/NF‐κB‐Associated Neuroinflammation and Modulates Inhibitory/Excitatory Synaptic Marker Expression After Spinal Cord Injury

**DOI:** 10.1002/cns.71086

**Published:** 2026-08-13

**Authors:** Wenjie Lu, Yurong Tu, Renkai Wang, Minghao Jiang, Junyu Zhuang, Jiahui Song, Ping Wu, Sunren Sheng, Sipin Zhu, Zhouguang Wang

**Affiliations:** ^1^ Department of Orthopaedics, The Second Affiliated Hospital and Yuying Children's Hospital of Wenzhou Medical University Wenzhou Medical University Wenzhou China; ^2^ State Key Laboratory of Macromolecular Drugs and Large‐Scale Preparation, School of Pharmaceutical Science Wenzhou Medical University Wenzhou China; ^3^ The Second Clinical School of Wenzhou Medical University Wenzhou China; ^4^ Oujiang Laboratory (Zhejiang Lab for Regenerative Medicine, Vision and Brain Health), School of Pharmaceutical Science Wenzhou Medical University Wenzhou China; ^5^ The First Affiliated Hospital of Wenzhou Medical University Wenzhou Medical University Wenzhou China

**Keywords:** Artemin‐Fc fusion protein, microglial polarization, neuropathic pain, spinal cord injury, TLR4/NF‐κB

## Abstract

**Aims:**

Spinal cord injury (SCI) can cause severe neurological dysfunction and the occurrence of chronic neuropathic pain, which can manifest as the occurrence of abnormal pain and hyperalgesia. Artemin (ARTN) is a member of the glial cell‐derived neurotrophic factor (GDNF) family ligand and can improve neural injury and regulate the occurrence of neuropathic pain. However, the process by which ARTN regulates inflammation and the sensitization of the dorsal horn of the spinal cord related to pain after SCI is still unclear.

**Methods:**

ARTN‐Fc fusion protein was constructed and administered intrathecally to mice after SCI. Motor recovery and pain‐related behaviors were evaluated using behavioral, gait, electrophysiological, paw withdrawal latency, and formalin‐induced Fos assays. Molecular changes were assessed by Western blotting, immunofluorescence, and immunohistochemistry. In vitro, a BV2–PC12 Transwell co‐culture system was used to examine the effect of ARTN‐Fc on activated microglia‐mediated neuronal injury.

**Results:**

ARTN‐Fc treatment significantly improved motor recovery and reduced thermal hyperalgesia after SCI. Mechanistically, ARTN‐Fc promoted microglial M2 polarization, inhibited TLR4/NF‐κB activation, suppressed pro‐inflammatory cytokine expression, and attenuated NLRP3 inflammasome/pyroptosis‐related signaling. In the spinal dorsal horn, ARTN‐Fc increased inhibitory GABAergic markers, including vGAT and GAD1, while reducing the excitatory marker vGluT2, suggesting altered inhibitory/excitatory synaptic marker expression. In vitro, ARTN‐Fc reduced neuronal apoptosis mediated by activated microglia.

**Conclusion:**

Taken together, the results suggest that ARTN‐Fc is a potential therapeutic agent for SCI repair and neuropathic pain treatment by inhibiting the TLR4/NF‐κB pathway to suppress neuroinflammation and modulating inhibitory/excitatory synaptic marker expression in the spinal dorsal horn.

## Graphical abstract

Intrathecal ARTN‐Fc treatment attenuates TLR4/NF‐κB‐associated neuroinflammation, promotes microglia/macrophage polarization towards an anti‐inflammatory phenotype, and improves functional recovery after spinal cord injury. ARTN‐Fc also modulates inhibitory/excitatory synaptic marker expression in the L4–L5 spinal dorsal horn, which may contribute to reduced hyperalgesia. The schematic was created with BioRender.

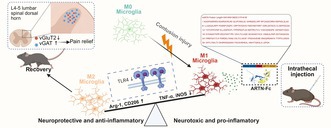

## 1 Introduction

Artemin (ARTN) is one of the glial cell line–derived neurotrophic factor (GDNF) family ligands of growth promoting peptides [1]. In cases of pathological nerve injury, ARTN exhibits a unique function in the development of neuropathic pain and changes in neural structure [2, 3]. Four receptors belonging to the GDNF family, namely GFRα1‐4, have been successfully identified. ARTN initially interacts with GFRα3, which is connected to the cell membrane through a glycosylphosphatidylinositol (GPI) anchor [4, 5]. By promoting the phosphorylation of intracellular tyrosine residues, ARTN‐GFRα3 facilitates the activation of RET, a receptor tyrosine kinase. This process plays a pivotal role in initiating essential pathways for sympathetic neuron migration, axon growth, and axon guidance [6, 7].

Primary damage in spinal cord trauma is attributed to mechanical impact, whereas secondary damage involves inflammation, apoptosis, oxidative stress, and other contributing factors. Severe functional impairment occurs as a consequence of extensive damage to numerous nerve cells caused by secondary injury [8, 9]. After SCI, microglia are rapidly activated and participate in neuroinflammatory regulation. Pro‐inflammatory M1‐like microglia release cytokines such as TNF‐α, IL‐1β, and IL‐6, thereby aggravating secondary injury, whereas anti‐inflammatory M2‐like microglia secrete factors such as IL‐10 and IL‐4, which may contribute to tissue repair and neuroregeneration [10, 11]. After spinal cord injury, there is a swift induction and accumulation of M1 microglia/macrophages near the location of the injury. The rise in the number of M2 microglia/macrophages during the initial phase was considerably less noticeable compared to that observed for the M1 phenotype [12, 13, 14].

In the central nervous system, Toll‐like receptor 4 (TLR4) is predominantly expressed on microglia [15]. TLR4, a receptor involved in the recognition of patterns, exhibits close association with innate immune responses and inflammatory processes. When TLR4 is stimulated by either endogenous or exogenous ligands, it initiates a cascade of signal transduction pathways, which includes the induction of the Nuclear factor kappa B (NF‐κB) pathway [16]. After its activation, NF‐κB relocates to the cell's nucleus, facilitating the upregulation of genes associated with inflammation and generating substantial quantities of cytokines that promote inflammatory responses [17]. NF‐κB activation also serves as an important priming signal for NLRP3 inflammasome activation. After SCI, activation of the NLRP3 inflammasome promotes caspase‐1 cleavage, GSDMD‐mediated pyroptosis, and IL‐1β maturation, thereby amplifying neuroinflammation and secondary injury [18]. Therefore, targeting the TLR4/NF‐κB/NLRP3 inflammatory axis may be beneficial for limiting excessive inflammation after SCI.

In addition, ARTN has been implicated in the regulation of neuropathic pain. Peripheral ARTN signaling can modulate pain‐related channels such as TRPV1/TRPA1 in DRG sensory neurons and contribute to nociceptor sensitization under inflammatory conditions [19, 20, 21]. In contrast, ARTN‐related interventions have also shown potential analgesic effects, as supported by BG00010 clinical studies and evidence that restoration of ARTN levels can reduce neuropathic pain and dorsal horn remodeling after nerve injury or SCI [22, 23, 24]. These findings suggest that the role of ARTN in pain regulation is context‐dependent, and its effects on SCI‐associated neuroinflammation and dorsal horn sensitization remain to be clarified.

Recombinant human ARTN was fused to the Fc region of immunoglobulin to improve its therapeutic potential by extending protein stability, promoting dimer formation, and facilitating receptor binding [25, 26]. Owing to their structural similarity to human IgG, Fc‐fusion proteins often exhibit improved pharmacokinetic properties and prolonged biological activity [27, 28]. Therefore, ARTN‐Fc was designed to enhance the stability and durability of ARTN‐based therapy. In this study, ARTN‐Fc was administered intrathecally to mice after SCI to evaluate its effects on spinal cord repair and neuropathic pain. We demonstrated that ARTN‐Fc promoted neurological recovery, attenuated neuroinflammation associated with TLR4/NF‐κB/NLRP3 signaling, and alleviated thermal hyperalgesia. In addition, ARTN‐Fc treatment was associated with altered inhibitory/excitatory synaptic marker expression in the lumbar spinal dorsal horn.

## 2 Methods

### 2.1 Materials

Fetal bovine Serum (FBS) and Dulbecco's modified Eagle's medium (DMEM) were obtained from Gibco (CA, USA). ARTN‐Fc and Fc fusion protein were bought from MerryBio (Nanjing, China). Lipopolysaccharide (HY‐D1056), TNF‐α (HY‐P1860) and BAY 11‐7082 (HY‐13453) were purchased from MedChemExpress (NJ, USA). Arg‐1 (93668S) was acquired from Cell Signaling Technology (MA, USA). TNF‐α (ab6671), GAP43 (ab75810), NF200 (ab4680), GFAP (ab7260), NeuN (ab279297), MBP (ab7349) and vGluT1 (ab180188) were supplied by Abcam (Cambridge, UK). Iba1 (sc32725) was obtained from Santa Cruz (CA, USA). iNOS (AF0199), MRC1 (DF4149), p‐NFκB p65 (AF2006), NFκB p65 (AF5006), c‐fos (AF5354), vGluT2 (DF13296) and Cleaved Caspase 1 (AF4005) were obtained from Affinity (OH, USA). GAD1 (10408‐1‐AP), NLRP3 (19771‐1‐AP) and GSDMD (20770‐1‐AP) were obtained from Proteintech (Wuhan, China). TLR4 (505258), vGAT (511071), β‐actin (R23613), Bcl‐2 (250198), Bax (R22708), IL‐1β (511369), HRP‐conjugated secondary antibodies against mouse and rabbit IgG and enhanced ECL chemiluminescence detection kit were obtained from ZenBioScience (Chengdu, China). Transwell cell culture chambers (12‐well, pore size 0.4 μm) were purchased from Corning (AZ, USA). Fluorescent probe DCFH‐DA (S0033S) and Enhanced Cell Counting Kit‐8 (C0042) were purchased from Beyotime (Shanghai, China). Modified Hematoxylin–Eosin (HE) Stain Kit (G1121) and Nissl Stain Kit (G1430) were purchased from Solarbio (Beijing, China). Pre‐adsorbed Donkey Anti‐Rabbit/Mouse IgG H&L antibodies labeled with Alexa Fluor 488, Alexa Fluor 555, and Alexa Fluor 647 were obtained from Abcam (Cambridge, UK).

### 2.2 Animal Model of SCI

Eighty adult C57BL/6 mice weighing 20–25 g were procured from the Experimental Animal Center of Wenzhou Medical University. All animal‐related procedures received approval from the Animal Care and Use Committee of Wenzhou Medical University (No. wydw2024‐0029). The C57BL/6 mice were administered 1% pentobarbital for anesthesia. The exposure of the spinal cord occurred following the extraction of the spinous and laminoid processes at T9‐T10. Afterwards, a contusion was induced by dropping a weight of 10 g from a height of 5 cm. The mice were administered intraperitoneal injections of cefazolin sodium at a concentration of 0.9% twice daily, and manual assistance was provided for urination every morning and evening. After surgery, the corresponding intrathecal injections of ARTN‐Fc (50 μg/kg), Fc (24 μg/kg), and saline were given according to the protocol for Monday, Wednesday, and Friday, for a total of six injections within two weeks.

### 2.3 Fos Expression in the Context of Formalin‐Induced Inflammation

In awake, freely moving mice, 50 μL of a 10% formalin solution were subcutaneously injected on the underside of both hind paws for the experiment. After a time interval of three hours following the injection, the spinal dorsal horn at the L4‐L5 level was extracted and immunofluorescence techniques were employed to identify Fos expression. Quantification was conducted on Fos‐expressing cells situated in the dorsal horn of the ipsilateral side, specifically those located above the central canal's horizontal intersection line.

### 2.4 Paw Withdrawal Reaction From Hot Pain in 49°C Water Bath

Mice were allowed to adapt to the laboratory setting for at least half an hour, the temperature maintained in the water bath was set at 49°C, and the latency to the first occurrence of a nociceptive response (e.g., licking, hind paw lifting, or jumping) was measured. If the animal did not show obvious nociceptive response, the time limit for termination was established as 20 s in order to prevent any harm to the experimental animal's tissues. For each individual mouse, the measurements were conducted in triplicate with a time interval of 30 min between assessments of the bilateral hind limbs.

### 2.5 Functional Behavior Evaluation

The motor function recovery of mice was assessed at various time points (1, 3, 7, 10, 14, 21, and 28 days post‐modeling) using the BMS score test. A track measuring 8 cm × 50 cm was constructed and a layer of white paper was applied as its covering. To ensure the suitability of mice in dark environments, the track was further overlaid with dark‐colored plastic sheeting to prevent any exposure to light. Mice, whose hind feet were stained red, were positioned at the initial location of the pathway with the objective of traversing from one end to the other. The footprints of mice produced on white paper were scanned and analyzed. The measures of the final result were acquired by three examiners who were impartial to the conditions of experimentation and conducted their assessments independently.

The evaluation of hindlimb behavior was based on the previously published research articles [29]. DeepLabCutGUl 2.3.5 was used for deep learning tracking of the video data (frame rate 120 fps, resolution 1280 × 720), and 5 key points including Crest, hip joint, knee joint, ankle joint and toe were manually labeled. The data with valid trajectories were selected for analysis after model training. The stick‐like view of the hindlimb and the angle changes of the hip joint, knee joint and ankle joint were drawn using MATLAB R2023b. The foot error during the gait cycle and the standing height were extracted, and the therapeutic effect was evaluated through one‐way analysis of variance (One‐way ANOVA).

### 2.6 Electrophysiological Evaluations of Hindlimb Motor Function

The animals were administered sodium pentobarbital (40 mg/kg, i.p.) for anesthesia. A needle electrode designed to provide stimulation (DSN1620, Medtronic, USA) was inserted subcutaneously at the nasal base with the tip in contact with the scalp as an anode. The needle electrode was positioned as a cathode at the midpoint of the subcutaneous ear. The gastrocnemius muscle was penetrated by the recording electrode, while a subcutaneous ground electrode was positioned at the tail's base. Electrical stimulation pulses (5 mA, 0.1 ms, 1 Hz) were applied to stimulate the brain using a stimulator (Keypoint, Medtronic, USA). Each mouse underwent three repetitions of electrical stimulation with intervals of 30 s between each repetition. The amplitude of every evoked response elicited by a solitary stimulus was recorded.

### 2.7 Hematoxylin & Eosin Staining and Nissl Staining

Twenty‐eight days post spinal cord injury (SCI), mice in each group were administered a lethal dose of sodium pentobarbital, followed by adequate cardiac perfusion with normal saline and 4% paraformaldehyde solution. The segment encompassing the lesion was embedded in paraffin. Paraffin sections 5 μm thick were attached to polylysine‐coated glass slides. After conventional dewaxing and hydration, HE staining mainly includes staining with hematoxylin staining solution for 3 min, differentiation solution for 5 s, blueing for 30 s, and then staining with eosin staining solution for 2 min. Nissl staining involves incubation with tar violet dye at 56°C for 1 h, differentiation with Nissl differentiation solution for 2 min until the background is almost colorless, and finally performing standard procedures of dehydration, xylene clearing and sealing. Images were observed and captured using digital slides scanning system (OLYMPUS, VS200, JPN).

### 2.8 Immunofluorescence Staining

Cellular immunofluorescence staining was performed to fix, permeabilize, and block the cells. Incubate the fixed cells on the slide with the primary antibody overnight at 4°C. Then, incubate with the corresponding secondary antibody for 1 h at 37°C while operating in a dark environment.

Tissue immunofluorescence staining: The spinal cords that were obtained were preserved in 4% paraformaldehyde for a duration of 48 h prior to being embedded in paraffin and subsequently sectioned. After the sections were dried, deparaffinized in xylene, and hydrated with gradient ethanol, the samples were rinsed three times with PBS. Subsequently, the tissues were subjected to incubation at 37°C for 1 h with a solution containing 5% BSA. This was followed by overnight incubation at 4°C with primary antibodies listed below. Subsequently, the sections were incubated with donkey anti‐rabbit/mouse secondary antibodies for 1 h at 37°C. Finally, the slices were sealed with DAPI‐containing anti‐fluorescence quench agent (Solarbio, Beijing, China). The fluorescence images were observed using digital slide scanning system (OLYMPUS, VS200, JPN) and laser confocal microscope (NIKON, C2si, JPN).

### 2.9 Immunohistochemistry Staining

Paraffin‐embedded sections were deparaffinized, rehydrated, and subjected to antigen retrieval with citrate buffer. After blocking endogenous peroxidase with 3% H_2_O_2_ and nonspecific binding with 5% BSA, sections were incubated with primary antibodies overnight at 4°C. The next day, sections were incubated with HRP‐conjugated secondary antibodies, visualized with DAB reagent (Zhongshan Golden Bridge, ZLI‐9017), counterstained with hematoxylin, dehydrated, mounted, and imaged under a bright‐field microscope.

### 2.10 Western Blot Analysis

Spinal cord tissues, including the injury site and L4–L5 spinal cord segments when indicated, were rapidly isolated and stored at −80°C. The tissue samples were subjected to homogenization using a modified radioimmunoprecipitation assay (RIPA) buffer. The protein levels were measured using bicinchoninic acid (BCA) reagents (Thermo, Rockford, IL, USA). The protein samples were loaded onto gels at a concentration of 40 μg per lane, followed by transfer onto a polyvinylidene fluoride (PVDF) membrane (Bio‐Rad, Hercules, CA, USA). The membrane was pre‐treated with blocking buffer (Beyotime, China) for 20 min, then incubated overnight at 4°C with the primary antibody. The membrane was then washed three times (10 min each) with TBST, followed by incubation with HRP‐conjugated secondary antibodies at room temperature for a duration of 2 h. Signals were detected by ImageQuant 800 (Amersham, USA) and band densities were measured and quantified by Image J software.

### 2.11 Cell Culture and Treatment

The microglia cell line BV2 and rat pheochromocytoma cell line PC12 were purchased from iCell (Shanghai, China). The cells were incubated at 37°C in a CO_2_ incubator with Dulbecco's modified Eagle medium supplemented with 10% fetal bovine serum and 1% penicillin–streptomycin.

For the BV2 cell viability assay, cells were seeded in 96‐well plates at a density of 1 × 10^4^ cells per well and cultured for 24 h. Cells were then treated with different concentrations of ARTN‐Fc (0, 252, 504, 1008, 1512, and 2016 ng/mL) for 24 h. Subsequently, CCK‐8 solution (10% v/v) was added for approximately 1 h, and the absorbance at 450 nm was measured using a microplate reader.

For the PC12 cell viability assay, PC12 cells were seeded in 96‐well plates at a density of 1 × 10^4^ cells per well. Cells were treated with TBHP (150 μM) together with different concentrations of ARTN‐Fc (0, 25.2, 252, 504, 756, 1008, 1260, and 1512 ng/mL) for 24 h. Cell viability was then assessed using the same CCK‐8 procedure.

For LPS‐induced inflammatory activation, BV2 cells were divided into six groups: Control, LPS, LPS + Fc, LPS + ARTN‐Fc, LPS + BAY 11–7082, and LPS + ARTN‐Fc + TNF‐α. Cells in the LPS group were treated with LPS (1 μg/mL) for 24 h. For pretreatment groups, cells were pretreated with Fc (600 ng/mL), ARTN‐Fc (504 ng/mL), or BAY 11–7082 (5 μM) for 2 h, followed by LPS stimulation for 24 h. For the NF‐κB reactivation experiment, BV2 cells were pretreated with ARTN‐Fc (504 ng/mL) for 2 h and then stimulated with LPS (1 μg/mL) plus TNF‐α (20 ng/mL) for 24 h. Control cells were cultured in fresh medium for 24 h. Cells were then collected for Western blotting and immunofluorescence analysis.

### 2.12 Co‐Culture of BV2 Microglia With Neuronal PC12 Cells

To evaluate the effect of ARTN‐Fc on LPS‐induced microglial inflammation on neuronal toxicity in vitro, BV2 cells and PC12 cells were co‐cultured using a 12‐well Transwell system (0.4 μm pore size). Before co‐culture, BV2 cells (5 × 10^4^ cells) were pretreated with ARTN‐Fc (504 ng/mL) and Fc (600 ng/mL) for 2 h, followed by stimulation with or without 1 μg/mL LPS for 24 h. After 24 h, BV2 cells were washed twice with PBS and then co‐cultured with PC12 cells (8 × 10^4^ cells) for 24 h. Immunofluorescence was used to detect neuronal apoptosis.

### 2.13 Intracellular ROS Detection

After the above processing, PC12 cells were incubated with fluorescent probe DCFH‐DA (10 μmol/L) in an incubator at 37°C for 20 min. PBS was washed 3 times, and ROS was detected by flow cytometry after digestion.

### 2.14 Statistical Analysis

All behavioral assessments and histological image quantifications were performed by investigators blinded to the experimental groups. The experimental results were represented as means ± SEM in the current format. ImageJ and GraphPad Prism 8 were utilized for statistical analysis. For comparisons among multiple groups, one‐way ANOVA followed by Tukey's multiple comparisons test was used. For behavioral data measured across multiple time points, two‐way repeated‐measures ANOVA followed by Tukey's multiple comparisons test was used to compare differences among groups at each time point. Statistical significance was determined at the *p < 0.05 and **p < 0.01 levels for all tests conducted.

## 3 Results

### 3.1 Regulation of ARTN‐Fc on LPS Induced M2/M1 Polarization of BV2 Cells

**FIGURE 1:**
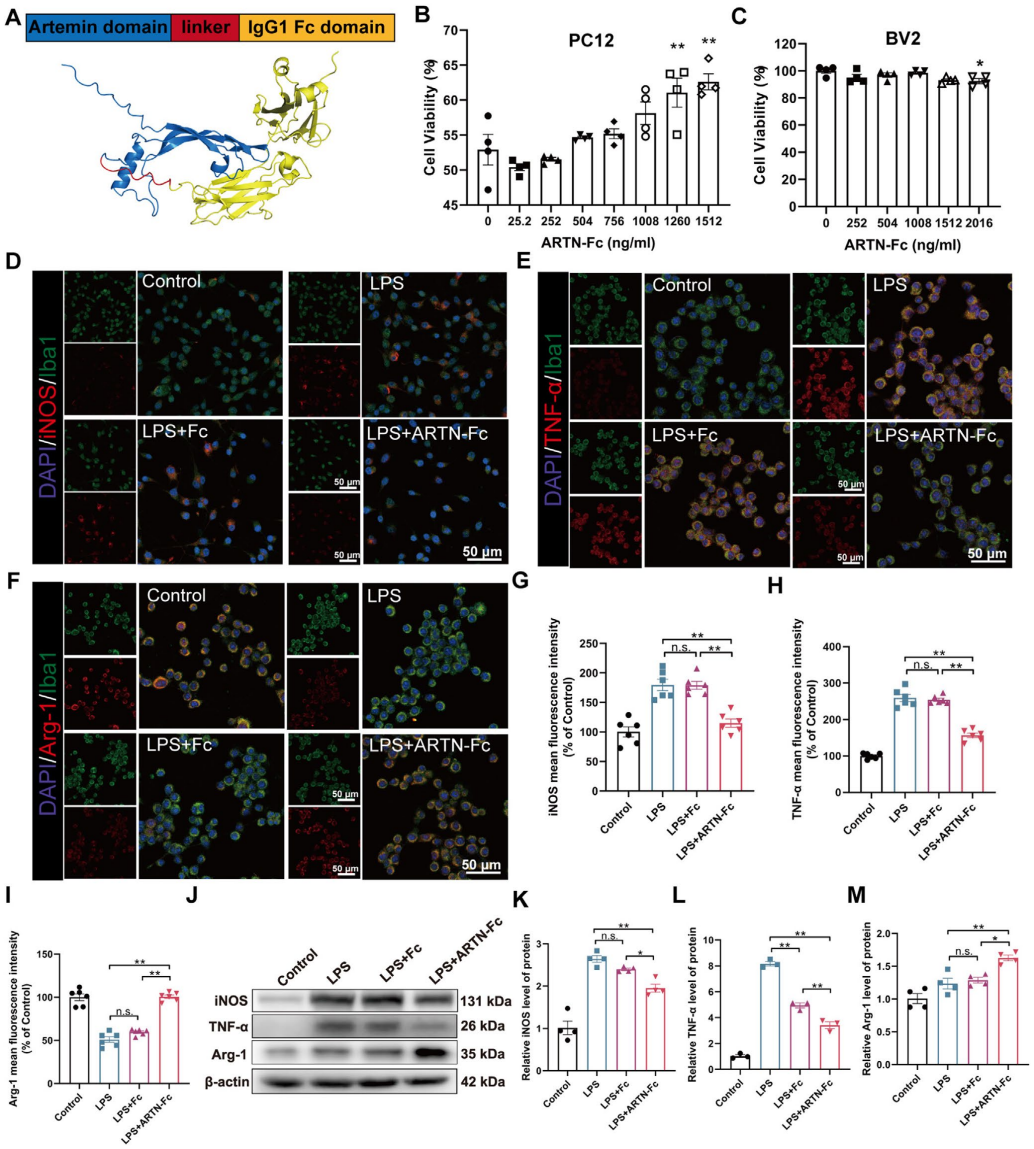
ARTN‐Fc promotes microglia M1 to M2 switch. (A) Schematic representation of the Artemin‐Fc fusion protein domain architecture. The fusion protein consists of: Artemin (ARTN) domain, IgG1 Fc domain and Linker sequence. (B) Under the TBHP stimulation concentration (150 μM), the PC12 cells were co‐cultured with different concentrations of ARTN‐Fc (0, 25.2, 252, 504, 756, 1008, 1260, 1512 ng/mL) for 24 h, and the cell viability was detected by CCK8 method. (C) Viability of BV2 cells measured by CCK‐8 assay after 24‐h incubation with increasing concentrations of ARTN‐Fc (0, 252, 504, 1008, 1512, 2016 ng/mL). (D–F) Immunofluorescence imaging showing the proportion of iNOS, TNF‐α and Arg‐1 positive BV2 cells cultured in each group. Green IF represents the microglia/macrophage specific protein marker Iba‐1, red fluorescence represents the M1/M2 microglia/macrophage phenotype marker iNOS, TNF‐α or Arg‐1, blue fluorescence represents the nuclear marker DAPI. Scale bars: 50 μm. (G–I) Quantification of fluorescence intensity of iNOS, TNF‐α and Arg‐1 level in each group (*n* = 6). (J) Western blotting performed for expression of iNOS, TNF‐α and Arg‐1 which was corrected by β‐actin as internal control. (K–M) Quantitative analysis of iNOS, TNF‐α and Arg‐1 protein expressions (*n* = 3). Statistical analysis was performed using one‐way ANOVA followed by Tukey's multiple comparisons test. *p < 0.05, **p < 0.01, n.s. = not significant.

To enhance the therapeutic potential of Artemin, we engineered a chimeric Artemin‐Fc fusion protein (ARTN‐Fc) (Figure 1A). This recombinant protein adopts a modular architecture consisting of three functional domains: an N‐terminal Artemin domain (ligand‐binding region), which retains the native neurotrophic activity through specific interactions with the GFRα3‐RET receptor complex; a flexible peptide linker, enabling spatial separation between domains to prevent steric interference; a C‐terminal human IgG1 Fc domain, facilitating dimerization via hinge‐region disulfide bonds and prolonging serum half‐life through FcRn‐mediated recycling. This design synergistically combines Artemin's neuronal regenerative properties with the Fc domain's pharmacokinetic advantages, thereby optimizing bioavailability for neural repair applications.

PC12 cells exposed to 150 μM tert‐butyl hydroperoxide (t‐BHP) exhibited significant cell death. Treatment with ARTN‐Fc at concentrations ranging from 0 to 1008 ng/mL failed to rescue this oxidative damage. It should be noted that a modest protective effect occurred only at the supraphysiological dose of 1260 ng/mL (Figure 1B). This narrow therapeutic window indicates that ARTN‐Fc requires exceptionally high concentrations (> 1 μg/mL) to exert marginal neuroprotective effects against acute oxidative stress in PC12 neurons. After 24‐h exposure to increasing ARTN‐Fc concentrations (0 to 2016 ng/mL) in BV2 cells, no significant toxicity was observed below 1512 ng/mL. Above the concentration of 2016 ng/mL, it showed a slight decrease in cell viability (Figure 1C). Therefore, in the present study, a concentration of 504 ng/mL ARTN‐Fc was used for subsequent experiments involving BV2 cells.

Microglia can change their morphology in response to the external environment. In response to inflammatory stimuli, microglia can be activated and acquire a M1‐like phenotype, forming a harmful neuronal microenvironment by producing inflammatory cytokines, enzymes that promote persistent tissue inflammation and reactive oxygen species (ROS) [12, 30]. We established regulators of the M1 phenotype of microglial by stimulating BV2 cells with Lipopolysaccharide (LPS). Immunofluorescence staining showed that ARTN‐Fc considerably reduced the M1 polarization of BV2 cells and reinforced the M2 polarization of BV2 cells by co‐culturing ARTN‐Fc with BV2 cells for 24 h. Meanwhile, LPS‐treated BV2 cells exhibited numerous lamellae and elongated filopodia, resembling microglia polarized towards the M1 phenotype. When ARTN‐Fc was co‐cultured with BV2 cells, the proportion of BV2 cells with lamellar processes significantly decreased, and most cells reverted to dense M2‐polarized microglia, while Fc fusion protein failed to alter the morphological changes brought about by LPS treatment (Figure 1D–I). In addition, the corresponding marker expression was using Western blot analysis. Compared with the LPS group, ARTN‐Fc significantly reduced the protein expression of M1 markers Inducible Nitric Oxide Synthase (iNOS) and Tumor Necrosis Factor‐alpha (TNF‐α), and increased the M2 marker Arginase‐1 (Arg‐1) (Figure 1J–M).

### 3.2 ARTN‐Fc Suppresses LPS‐Induced TLR4/NF‐κB Signaling Activation in BV2 Cells

**FIGURE 2:**
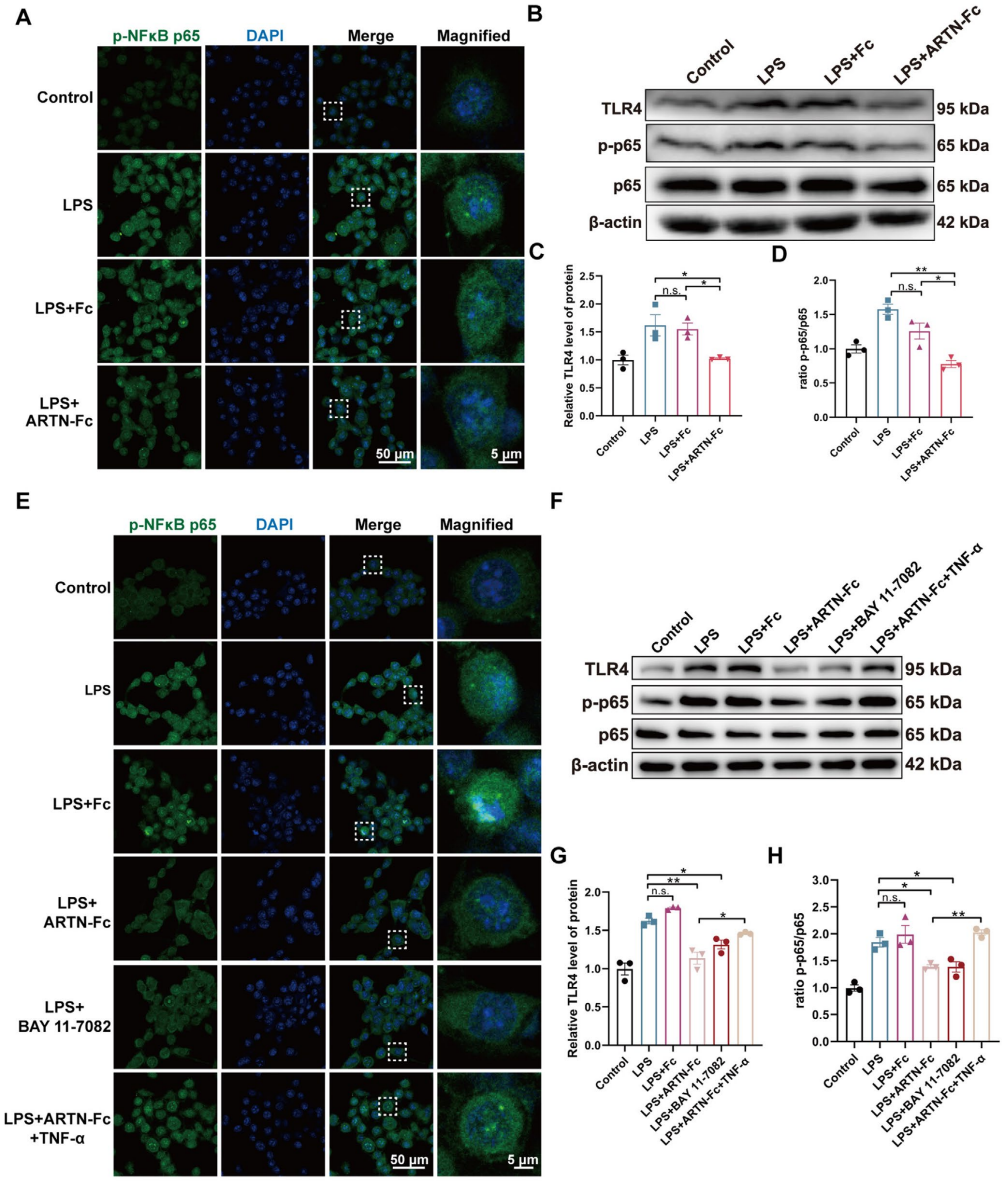
Effect of ARTN on the nuclear translocation of p‐NF‐κB p65 in LPS‐stimulated BV‐2 cells. (A) BV‐2 cells were stimulated with LPS (1 μg/mL) in the absence or presence of ARTN for 24 h, followed by detection of the p‐NF‐kB p65 subunit translocation by immunofluorescence. p‐NF‐κB p65 is shown in green, blue fluorescence represents the nuclear marker DAPI. Scale bar = 50 μm. (A boxed region illustrates a representative region with high power images, scale bar = 5 μm) (B) BV‐2 cells were stimulated with LPS (1 μg/ml) in the absence or presence of ARTN for 24 h, and TLR4, p‐NF‐κB p65 and NF‐κB p65 levels were determined by Western blot. β‐actin were used as endogenous controls. (C, D) Quantitative analysis of TLR4, p‐NF‐κB p65 and NF‐κB p65 protein expressions (*n* = 3). (E) BV2 cells were treated with LPS, Fc, ARTN‐Fc, BAY 11‐7082, or ARTN‐Fc combined with TNF‐α, followed by immunofluorescence staining for p‐NF‐κB p65. p‐NF‐κB p65 is shown in green, and nuclei were counterstained with DAPI (blue). Scale bar = 50 μm; magnified images, scale bar = 5 μm. (F) Representative Western blot images showing the protein levels of TLR4, p‐NF‐κB p65, and NF‐κB p65 in BV2 cells from each group. β‐actin was used as an endogenous control. (G, H) Quantitative analysis of TLR4 protein expression and the p‐NF‐κB p65/NF‐κB p65 ratio (*n* = 3). Statistical analysis was performed using one‐way ANOVA followed by Tukey's multiple comparisons test. *p < 0.05, **p < 0.01, n.s. = not significant.

Toll‐like receptors (TLRs), as pattern recognition receptors, are recognized by microglia and closely linked to the regulation of the inflammatory response [31]. Nuclear factor‐κB (NF‐κB) is located downstream of TLR4 signaling pathway [32]. Lipopolysaccharide (LPS) has the ability to induce TLR4 activation in vivo, leading to cellular harm. Subsequently, NF‐κB relocates to the nucleus and modulates the expression of inflammatory genes associated with this process [33]. Immunofluorescence staining of p‐NFκB p65 was used to observe its translocation in the nucleus after ARTN‐Fc administration. Repositioning of p‐NFκB p65 into the nucleus can be triggered by LPS, and the intervention of ARTN‐Fc can reduce the nuclear translocation of p‐NFκB p65 and play an anti‐inflammatory role. However, Fc fusion protein group had no inhibitory effect on LPS‐induced nuclear translocation (Figure 2A).

Moreover, Western blot analysis unveiled a remarkable increase in expression levels of p‐NFκB p65 and TLR4 in the LPS group, which was effectively attenuated by ARTN‐Fc treatment (Figure 2B–D). However, administration of Fc fusion protein failed to mitigate the LPS‐induced increase in p‐NFκB p65 and TLR4 expression.

To further verify the involvement of NF‐κB signaling, BV2 cells were treated with BAY 11‐7082 or TNF‐α in the presence of LPS and ARTN‐Fc. Immunofluorescence staining showed that LPS markedly increased p‐NFκB p65 activation and nuclear accumulation, which was not inhibited by Fc treatment. ARTN‐Fc reduced LPS‐induced p‐NFκB p65 activation, and a similar effect was observed after BAY 11‐7082 treatment. In contrast, TNF‐α partially restored p‐NFκB p65 activation in ARTN‐Fc‐treated cells (Figure 2E).

Western blot analysis further showed that LPS increased TLR4 expression and the p‐p65/p65 ratio. These changes were not significantly reversed by Fc, but were reduced by ARTN‐Fc treatment. BAY 11‐7082 also suppressed NF‐κB activation, whereas TNF‐α partially restored p‐p65/p65 levels in the presence of ARTN‐Fc. These results suggest that inhibition of TLR4/NF‐κB signaling contributes, at least in part, to the anti‐inflammatory effect of ARTN‐Fc in LPS‐stimulated BV2 cells (Figure 2F–H).

Consistent with these findings, immunofluorescence staining of inflammatory phenotype markers showed that LPS increased iNOS expression and reduced Arg‐1 expression in BV2 cells. ARTN‐Fc and BAY 11‐7082 both decreased iNOS staining and enhanced Arg‐1 staining, whereas TNF‐α partially reversed the regulatory effect of ARTN‐Fc on these markers (Figure S1A–D). Western blot analysis showed a similar trend, with decreased iNOS and increased Arg‐1 expression after ARTN‐Fc or BAY 11–7082 treatment, while TNF‐α partially abolished these effects (Figure S1E–G). These results further support that ARTN‐Fc suppresses LPS‐induced pro‐inflammatory activation and promotes an anti‐inflammatory phenotype in BV2 cells, at least partly through inhibition of NF‐κB signaling.

### 3.3 ARTN‐Fc Attenuates Neuronal Apoptosis Mediated by LPS‐Activated BV2 Cells

**FIGURE 3:**
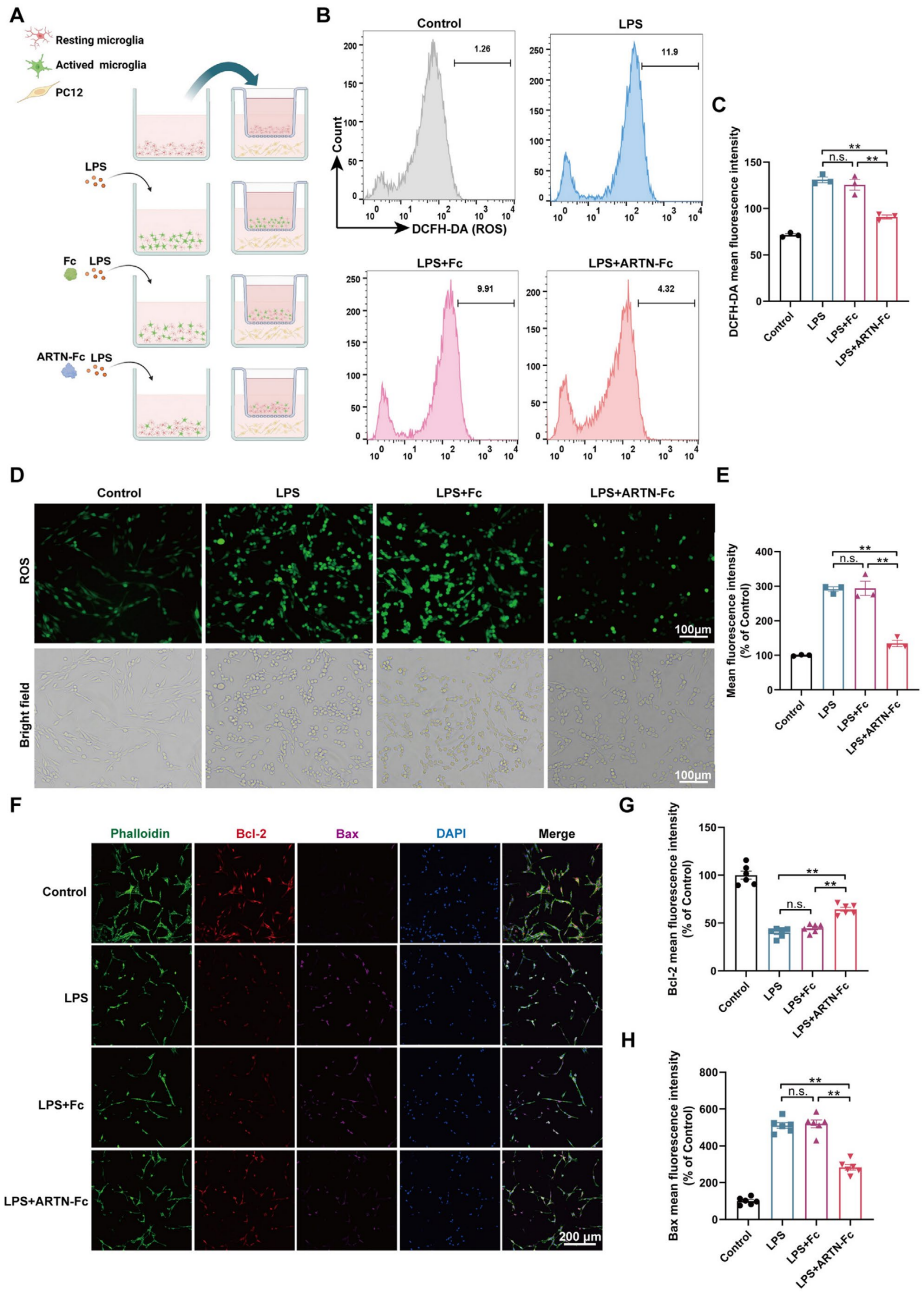
Neuroprotective effect of ARTN‐Fc on LPS‐stimulated activated microglia mediated neurotoxicity. (A) Schematic representation of the co‐culture of BV2 and PC12 cells. (B, C) Intracellular ROS were detected by flow cytometry. The mean fluorescence intensity was quantitatively analyzed (*n* = 3). (D, E) Intracellular ROS was detected under a microscope and its mean fluorescence intensity was quantified (*n* = 3). Scale bars: 100 μm. (F) Representative immunofluorescence images showing PC12 cell morphology labeled with phalloidin (green), together with Bcl‐2 (red) and Bax (orange) expression. Scale bar: 200 μm. (G, H) Quantitative analysis of Bcl‐2 and Bax fluorescence intensity in PC12 cells (*n* = 6). Statistical analysis was performed using one‐way ANOVA followed by Tukey's multiple comparisons test. **p < 0.01, n.s. = not significant.

To evaluate the effect of ARTN‐Fc on neuronal apoptosis induced by neuroinflammation in vitro, we performed co‐culture experiments of BV2 and PC12 cells via transwell chambers (Figure 3A). The results demonstrated that when PC12 cells were co‐cultured with untreated BV2 cells, intracellular reactive oxygen species (ROS) levels remained low. However, PC12 cells co‐cultured with lipopolysaccharide (LPS)‐treated BV2 cells exhibited a significant increase in ROS production. Notably, pretreatment with ARTN‐Fc effectively suppressed intracellular ROS levels in PC12 cells under these conditions. In contrast, the Fc group did not show a significant inhibitory effect on ROS accumulation (Figure 3B,C). The same trend was also observed in ROS fluorescence intensity detected by immunofluorescence (Figure 3D,E). Bax is a pro‐apoptotic gene, while Bcl‐2 is an anti‐apoptotic gene. In order to determine whether ARTN‐Fc could inhibit neuronal apoptosis, cytoskeleton staining of PC12 cells was performed with phalloidin. The results showed that the cells in the Control group had complete cell morphology, increased synapses, elongated axons, and reticular structures formed between cells. However, pretreatment with ARTN‐Fc could partially inhibit the above changes in PC12 cell morphology. The results showed that the fluorescence intensity of anti‐apoptotic Bcl‐2 in LPS group and LPS + Fc group was significantly lower than that in Control group, and the fluorescence intensity of pro‐apoptotic gene Bax in LPS group and LPS + Fc group was significantly higher than that in Control group. However, this phenomenon was reversed in the ARTN‐Fc group, which reduced the level of pro‐apoptosis and enhanced the ability of anti‐apoptosis (Figure 3F–H). Taken together, the data confirmed that ARTN‐Fc exerted a neuroprotective effect by inhibiting neuronal ROS production and apoptosis mediated by activated microglia.

### 3.4 The ARTN‐Fc Treatment Improved Motor Function Recovery in Mice With SCI

Before the long‐term in vivo experiments, a preliminary dose‐exploration assay was performed to select an appropriate dose of ARTN‐Fc. After 7 days of treatment, Western blot analysis showed that SCI increased iNOS, TLR4 and p‐p65/p65 levels, while reducing Arg‐1 expression. ARTN‐Fc treatment reduced iNOS, TLR4 and p‐p65/p65 levels and increased Arg‐1 expression, with 50 μg/kg producing a clear anti‐inflammatory effect. Since 100 μg/kg did not show an obvious additional benefit compared with 50 μg/kg, ARTN‐Fc at 50 μg/kg was selected for subsequent in vivo experiments (Figure S2A–F).

**FIGURE 4:**
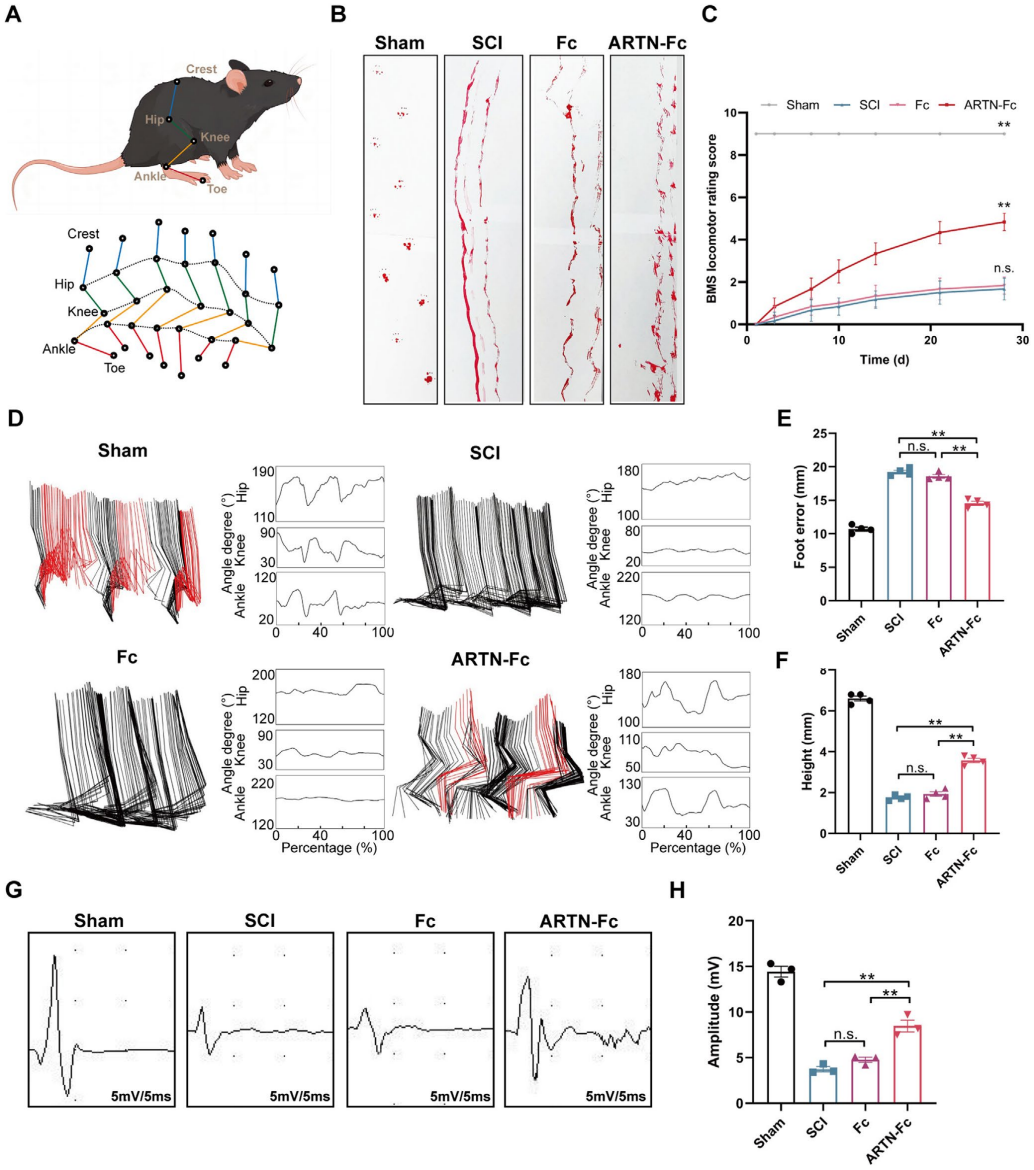
ARTN‐Fc promotes the recovery of motor function in mice with SCI. (A) Diagram of the hind limbs movement joints. (B) The gait marks of the mice in each group at Day 28. (C) BMS scores were performed on mice at the corresponding time points. (D) Color‐coded stick views of Sham, SCI, Fc, and ARTN‐Fc groups as well as angle curves of hip, knee, and ankle joints. (E, F) Statistical analysis of hind limb motor function of the mice, including (E) foot error and (F) height (*n* = 4). (G) Representative MEPs recorded from each group. (H) The amplitude was quantified and statistically analyzed (*n* = 3). BMS scores were analyzed by two‐way repeated‐measures ANOVA, and other multiple‐group data were analyzed by one‐way ANOVA, both followed by Tukey's multiple comparisons test. **p < 0.01, n.s. = not significant.

The neurotrophic factor ARTN‐Fc has been demonstrated to promote site‐specific regeneration of myelinated and unmyelinated sensory nerve axons, as well as sustained recovery of electrophysiological function [7, 34]. In order to achieve this, we administered intrathecal ARTN‐Fc for a duration of 2 weeks (3 times per week on Monday, Wednesday, and Friday) after spinal cord injury (SCI). Motor function in mice was assessed on day 1, 3, 7, 10, 14, 21, and 28. Colored stick diagrams simulate the positional connections of crest, hip, knee, ankle, and toe in mice (Figure 4A). The footprint imprinting experiment revealed that both the SCI group and the Fc fusion protein group exhibited significant disruption in waveform and dragging movement. After ARTN‐Fc treatment, despite the disarray of the footprints, a distinct lift‐off movement of the hind limbs was observed (Figure 4B). Comparatively higher BMS scores were noted in ARTN‐Fc‐treated animals compared to the SCI group (Figure 4C). Hindlimb movements were depicted in color‐coded stick plots. The hind leg joints of mice in the SCI and Fc fusion protein group exhibited a lack of flexion, with minimal joint mobility. In contrast, ARTN‐Fc‐treated mice exhibited mobile hind limb joints that occasionally supported their body weight. The amplitude of crests and toes in ARTN‐Fc group was higher than that in other groups, and the angle amplitude of hip, knee and ankle joints in ARTN‐Fc group was the greatest changed (Figure 4D). Quantitative analysis revealed that ARTN‐Fc‐treated mice displayed a more effective range of plantar support (Figure 4E) and achieved greater maximum height above ground level (Figure 4F) compared to both SCI and Fc fusion protein group, indicating an improved motor function in their hind limbs.

To investigate the function of the descending pathway responsible for transmitting nerve impulses from the lesion area to hindlimb motor neurons, we conducted motor evoked potential (MEP) recordings at 4 weeks post‐lesion. MEPs, which reflect neuromuscular unit connectivity, were recorded in the gastrocnemius muscle following electrical stimulation. In the Sham group, typical MEP waveforms were observed. However, after spinal cord injury, MEPs were significantly diminished, indicating disruption of neuromuscular units (Figure 4G). Interestingly, ARTN‐Fc‐treated mice exhibited greater amplitude compared to SCI mice, while no statistical difference was observed in the Fc group (Figure 4H). These findings suggest that ARTN‐Fc may further enhance recovery of bioelectrical conduction function following SCI.

### 3.5 ARTN‐Fc Promoted Tissue and Nerve Repair After Spinal Cord Injury

**FIGURE 5:**
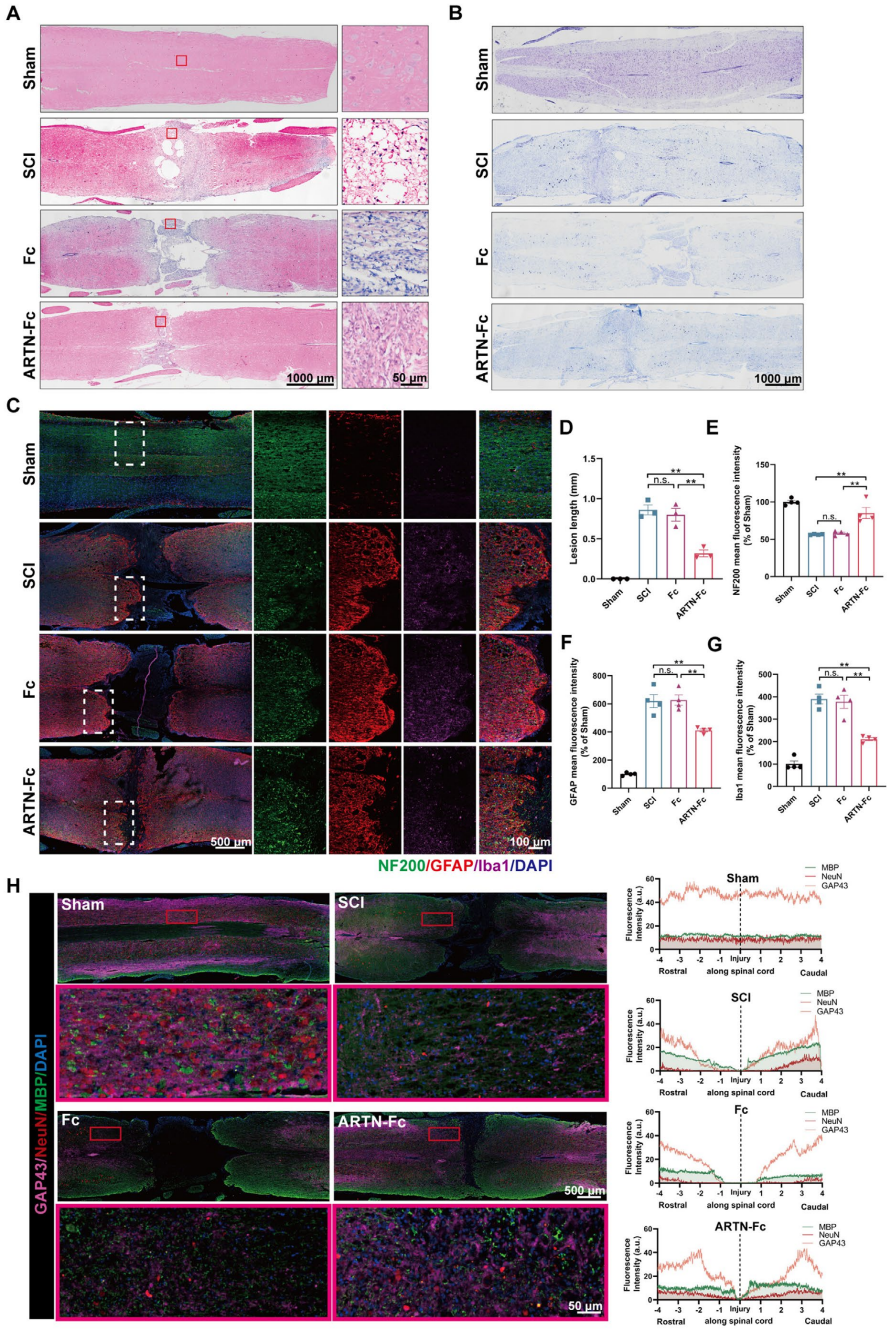
ARTN‐Fc promotes tissue and nerve repair after spinal cord injury. (A) H&E staining (longitudinal section) results for each group, scale bar = 1000 μm. A boxed region illustrates a representative region with high power images, scale bar = 50 μm. (B) Nissl staining of the different groups, scale bar = 1000 μm. (C) Representative immunofluorescence staining results of NF200, GFAP and Iba1 on a spinal cord section 28 days after spinal cord injury. The bright green dots are regeneration of nerves stained with NF200 positive. Bright red dots are thought to be glial scars with GFAP positive staining. Bright purple Iba1‐positive staining represents macrophages/microglia. Nuclei are marked with DAPI (blue), scale bar =500 μm. A boxed region illustrates a representative region with high power images, scale bar = 100 μm. (D) Quantitative analysis of lesion length (*n* = 3). (E–G) Quantitative analysis of immunofluorescence intensity for NF200, GFAP, and Iba1 in spinal cord tissue across experimental groups after spinal cord injury (*n* = 4). (H) The immunofluorescence staining results of GAP43 (purple), NeuN (red) and MBP (green). The nuclear is labeled by DAPI (blue). Scale bar: 500 μm. High‐magnification images of the boxed areas are shown in the inserts (ROI); scale bar: 50 μm. Intensity traces are plotted on the right. Statistical analysis was performed using one‐way ANOVA followed by Tukey's multiple comparisons test. **p < 0.01, n.s. = not significant.

After 28 days of treatment, HE staining was used to observe the histomorphology of the injured spinal cord. There was a significant void at the location of spinal cord injury in both the SCI and Fc fusion protein cohorts. However, the formation of cavities in the injured spinal cord tissue area was significantly reduced after ARTN‐Fc treatment (Figure 5A). Nissl staining was used to reflect the ability of nerve cells to synthesize proteins. We found that Nissl bodies were relatively more pronounced and abundant bilaterally in the injured area in the ARTN‐Fc‐treated group (Figure 5B).

Immunofluorescence staining was conducted to assess the growth of axons and inflammation at the site of injury, using NF‐200 (a marker for differentiated neurons), GFAP (a marker for reactive astrocytes), and Iba‐1 (a marker for microglia/macrophages). The results showed that the axons in the SCI group and Fc fusion protein group were almost broken and atrophied, and the scar boundary was obvious, while the axons in the ARTN‐Fc treatment group were significantly improved and extended to the scar boundary. In addition, the activated microglia in the SCI group and Fc fusion protein group were widely distributed in the injury site, while the microglia in the ARTN‐Fc group were less and scattered (Figure 5C–G).

Surviving neurons after spinal cord injury have a significant impact on the recovery of motor function. Regenerating neurons, growing axons and myelin sheathe were labeled with Neun, GAP43 and MBP, respectively. According to the fluorescence intensity curve analysis of MBP, NeuN, GAP43 along the rostral to caudal section of the spinal cord, the ARTN‐Fc group exhibited a significantly greater quantity of neurons and axons compared to both the SCI group and the Fc group, and there was a tendency to reduce demyelination (Figure 5H). In summary, the findings of this investigation suggest that ARTN‐Fc intervention can promote the recovery of motor function after SCI and has a clear neuroprotective effect.

### 3.6 ARTN‐Fc Promotes the Anti‐Inflammatory Phenotype Transformation of Microglia/Macrophages After Spinal Cord Injury

**FIGURE 6:**
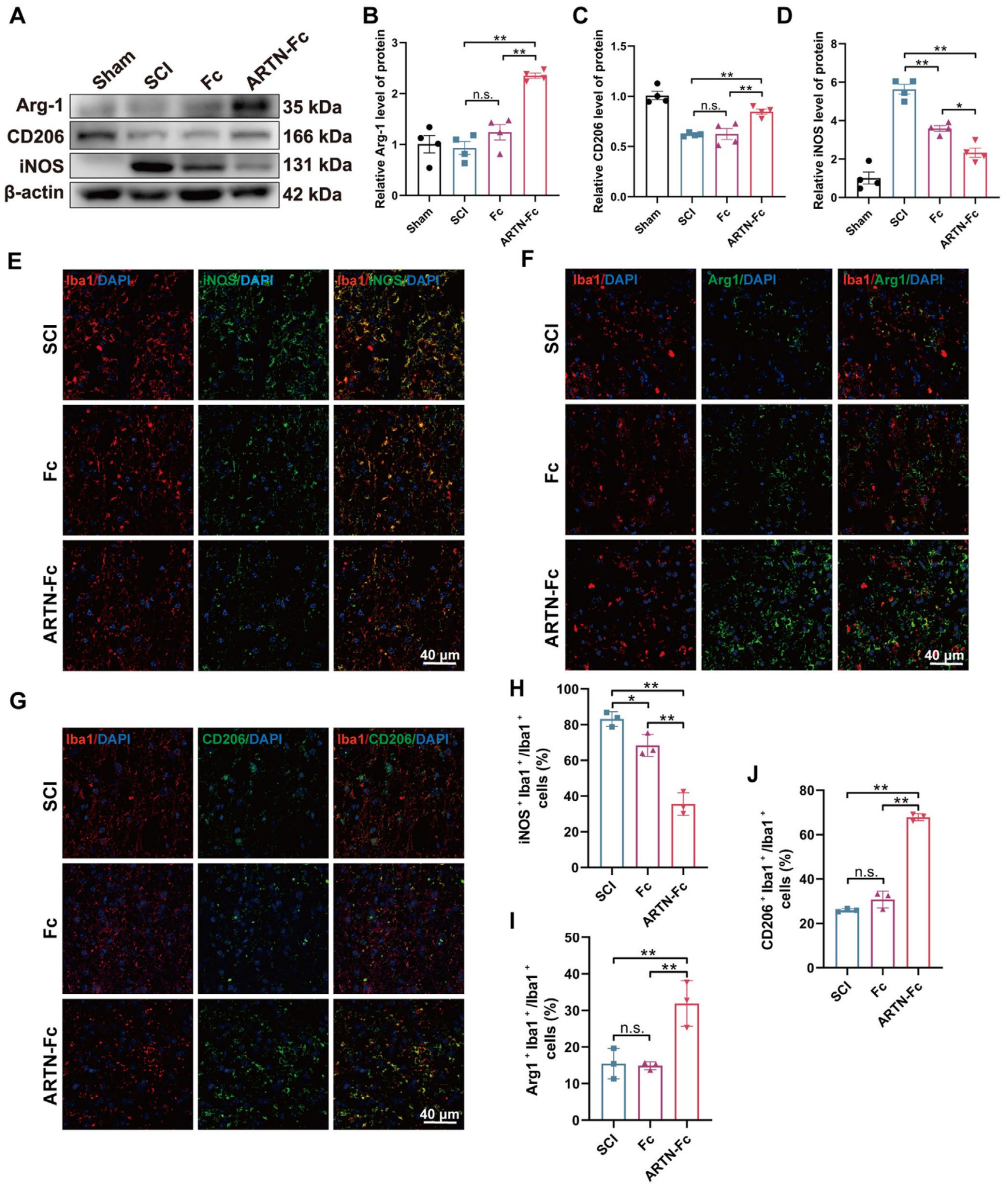
The administration of ARTN‐Fc following SCI facilitated the transition of microglia/macrophage polarization from M1 to M2 phenotype in vivo. (A) Western blotting performed for expression of Arg‐1, CD206 and iNOS, which was corrected by β‐actin as internal control. (B–D) Quantitative analysis of Arg‐1, CD206 and iNOS protein expressions (*n* = 4). (E–J) Representative immunofluorescence staining of Iba1 (red) and iNOS/Arg1/CD206 (green) in the injured spinal cord lesion areas and the analysis of iNOS^+^/Iba1^+^, Arg1^+^ /Iba1^+^ and CD206^+^ /Iba1^+^ microglia/macrophage cells in the traumatic lesion area (*n* = 3). Statistical analysis was performed using one‐way ANOVA followed by Tukey's multiple comparisons test. *p < 0.05, **p < 0.01, n.s. = not significant.

After spinal cord injury, microglia/macrophages become activated and polarized towards the M1 phenotype, resulting in tissue inflammation and exacerbation of neurological symptoms [35]. Therefore, enhancing the recovery process after SCI could be achieved by promoting the transformation of activated microglia/macrophages into an M2 phenotype with anti‐inflammatory properties. Western blot analysis was performed to detect the M1 phenotypic marker iNOS and the M2 phenotypic markers Arg‐1 and CD206 (Figure 6A–D). Compared with the SCI group, ARTN‐Fc treatment significantly reduced iNOS expression and increased Arg‐1 and CD206 expression in spinal cord tissues. Notably, Fc treatment also partially reduced iNOS expression compared with the SCI group, but it did not significantly increase Arg‐1 or CD206 expression. These results suggest that Fc may exert a limited effect on pro‐inflammatory marker expression, whereas ARTN‐Fc more effectively promotes an anti‐inflammatory phenotypic shift.

Apart from Western blot, immunofluorescence staining further confirmed the regulatory effect of ARTN‐Fc on microglial polarization. iNOS, Arg‐1, CD206, and Iba‐1 were analyzed by double immunofluorescence staining (Figure 6E–G). Compared with the SCI group, the proportion of iNOS^+^/Iba‐1^+^ cells was decreased in ARTN‐Fc mice on the 28th day after SCI (SCI 83.18% ± 2.37% vs. ARTN‐Fc 35.52% ± 3.67%, *p* < 0.01, Figure 6H), Arg‐1^+^/Iba‐1^+^ and CD206^+^/Iba‐1^+^ cells increased after spinal cord injury (SCI 15.45% ± 2.39% vs. ARTN‐Fc 31.89% ± 3.59%, *p* < 0.01, Figure 6I; SCI 25.92% ± 0.44% vs. ARTN‐Fc 67.84% ± 0.91%, *p* < 0.01, Figure 6J). The Fc fusion protein group only showed a decrease in the expression of pro‐inflammatory marker iNOS (SCI 83.18% ± 2.37% vs. Fc 68.42% ± 3.53%, *p* < 0.05, Figure 6H), but no increase in the expression of anti‐inflammatory markers Arg‐1 and CD206. The results collectively suggest that ARTN‐Fc promotes the transformation of microglia/macrophages into anti‐inflammatory phenotypes after SCI.

### 3.7 ARTN‐Fc Suppresses TLR4/NF‐κB Activation and Downstream NLRP3 Inflammasome‐Related Signaling After SCI

**FIGURE 7:**
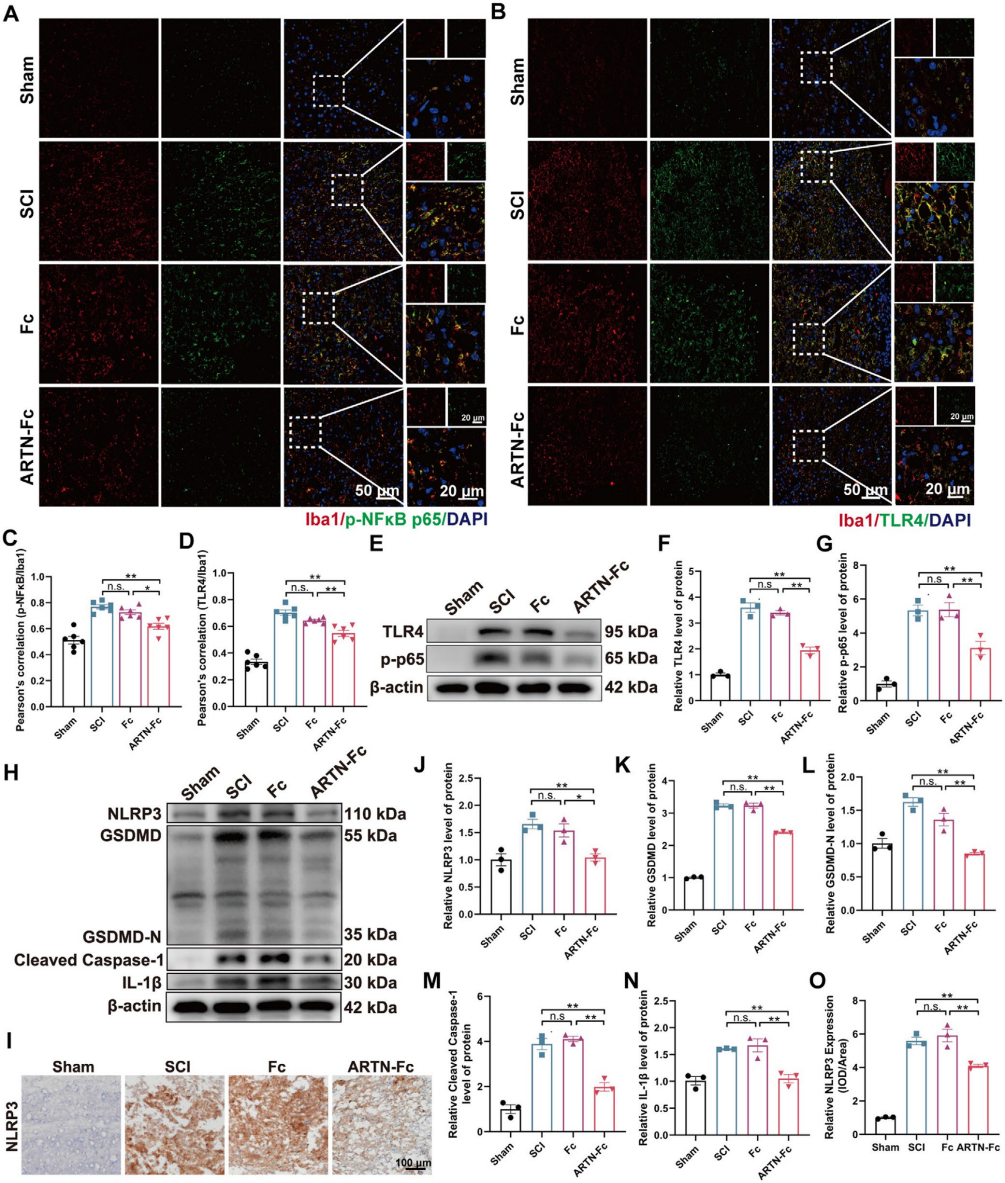
ARTN‐Fc regulates microglial M1/M2 polarization through TLR4/NF‐κB signaling cascades. (A, B) Representative immunofluorescence staining of Iba1 (red) and p‐NFκB/TLR4 (green) in the injured spinal cord lesion areas. The nuclei are labeled by DAPI (blue). Scale bar: 50 μm. High‐magnification images of the boxed areas are shown in the inserts (ROI); scale bar: 20 μm. (C, D) Colocalization analysis by Pearson's correlation. The colocalization of Iba1/p‐NFκB and Iba1/TLR4 as shown was estimated by calculating the Pearson's correlation of their fluorescence intensities (*n* = 6). (E) Western blotting performed for expression of TLR4 and p‐p65, which was corrected by β‐actin as internal control. (F, G) Quantitative analysis of TLR4 and p‐p65 protein expressions (*n* = 3). (H) Representative Western blot images showing the expression of NLRP3, GSDMD, GSDMD‐N, Cleaved Caspase‐1, and IL‐1β in spinal cord tissues. β‐actin was used as an endogenous control. (I) Representative immunohistochemical staining of NLRP3 in the injured spinal cord lesion area. Scale bar = 100 μm. (J–N) Quantitative analysis of NLRP3, GSDMD, GSDMD‐N, Cleaved Caspase‐1, and IL‐1β protein expression levels (*n* = 3). (O) Quantitative analysis of relative NLRP3 expression based on IOD/Area in immunohistochemical staining images (*n* = 3). Statistical analysis was performed using one‐way ANOVA followed by Tukey's multiple comparisons test. *p < 0.05, **p < 0.01, n.s. = not significant.

Given the crucial role of NF‐κB signaling in mediating the inflammatory response, we subsequently investigated the impact of ARTN‐Fc on TLR4/NF‐κB activation following SCI. We performed double immunostaining for Iba‐1 and p‐NFκB p65 as well as TLR4, respectively. In comparison to the Sham group, microglial cells exhibited heightened activation of the TLR4/NF‐κB signaling pathway subsequent to SCI. Immunofluorescence co‐staining of Iba‐1 and p‐NFκB p65 in the spinal cord injury area showed that the co‐localization degree of Iba‐1 and p‐NFκB p65 in the ARTN‐Fc group was lower than that in the SCI group (ARTN‐Fc 0.618 ± 0.023 vs. SCI 0.768 ± 0.016, *p* < 0.01; Determined by the mean Pearson's coefficient values; Figure 7A,C), while the Fc fusion protein group did not reach statistical significance in the degree of colocalization with the SCI group. The Toll‐like receptor 4 (TLR4) serves as a crucial upstream regulator of immune response, exerting its effects by modulating the expression of downstream signaling molecules such as NF‐κB. Immunofluorescence co‐staining involving Iba‐1 and TLR4 yielded consistent findings (ARTN‐Fc 0.548 ± 0.023 vs. SCI 0.703 ± 0.020, *p* < 0.01; Determined by the mean Pearson's coefficient values; Figure 7B,D). Consistently, Western blot analysis showed that SCI increased TLR4 expression and the p‐p65/p65 ratio, both of which were reduced after ARTN‐Fc treatment but not after Fc treatment (Figure 7E–G).

Since NF‐κB activation is closely associated with the priming of inflammasome‐related inflammatory responses, we further examined NLRP3 inflammasome‐ and pyroptosis‐related proteins in injured spinal cord tissues. Western blot analysis showed that SCI increased the expression of NLRP3, GSDMD, GSDMD‐N, Cleaved Caspase‐1, and IL‐1β. In contrast, ARTN‐Fc treatment markedly reduced the expression of these proteins, whereas Fc treatment did not show a comparable inhibitory effect (Figure 7H,J–N). Immunohistochemical staining further confirmed that NLRP3 expression was increased in the SCI group and was reduced after ARTN‐Fc treatment (Figure 7I,O). These results suggest that ARTN‐Fc suppresses TLR4/NF‐κB activation and attenuates downstream NLRP3 inflammasome/pyroptosis‐related inflammatory signaling after SCI.

### 3.8 ARTN‐Fc Alleviated Inflammatory Pain and Thermal Pain After Spinal Cord Injury

The Fos protein is expressed in spinal dorsal horn neurons and the activation of the proto‐oncogene c‐fos will occur promptly following the application of harmful stimuli. Therefore, mapping Fos expression in these neurons can effectively localize the optimal marker of neuronal populations that respond to noxious inputs in awake animals [36]. The L4‐L5 segment of the spinal cord was extracted from mice following a subcutaneous injection of 10% formalin on the surface of both hind paws, and immunofluorescence was employed to detect c‐fos expression in the dorsal horn of the spinal cord. The findings revealed an increased number of c‐fos^+^ neurons in the lumbar spinal cord's dorsal horn among mice in both the SCI group and Fc group compared to the sham group, with a subsequent decrease observed after ARTN‐Fc treatment (Figure S3A,B). Additionally, paw withdrawal latency was measured when subjecting mice's hind paws to harmful thermal stimulation by immersing them in a water bath at 49°C. Notably, compared to the SCI group, significant prolongation of paw withdrawal latency was observed for both left and right hind paws in the ARTN‐Fc group (Figure S3C). Consequently, continuous administration of ARTN‐Fc is suggested as a potential alleviator for pain behavior in mice experiencing persistent pain.

### 3.9 ARTN‐Fc Alleviates Hyperalgesia in Association With Altered Inhibitory/Excitatory Synaptic Marker Expression

In the behavioral study, we observed that both the SCI group and Fc fusion protein group exhibited stronger nociceptive responses and higher Fos expression during formalin‐induced inflammation. These groups also showed shortened paw withdrawal latency to noxious heat stimulation, whereas ARTN‐Fc treatment alleviated heat and inflammatory pain. Since chronic pain is closely associated with altered excitatory and inhibitory signaling in the spinal dorsal horn, we examined inhibitory and excitatory synaptic marker expression in the L4–L5 dorsal horn.

**FIGURE 8:**
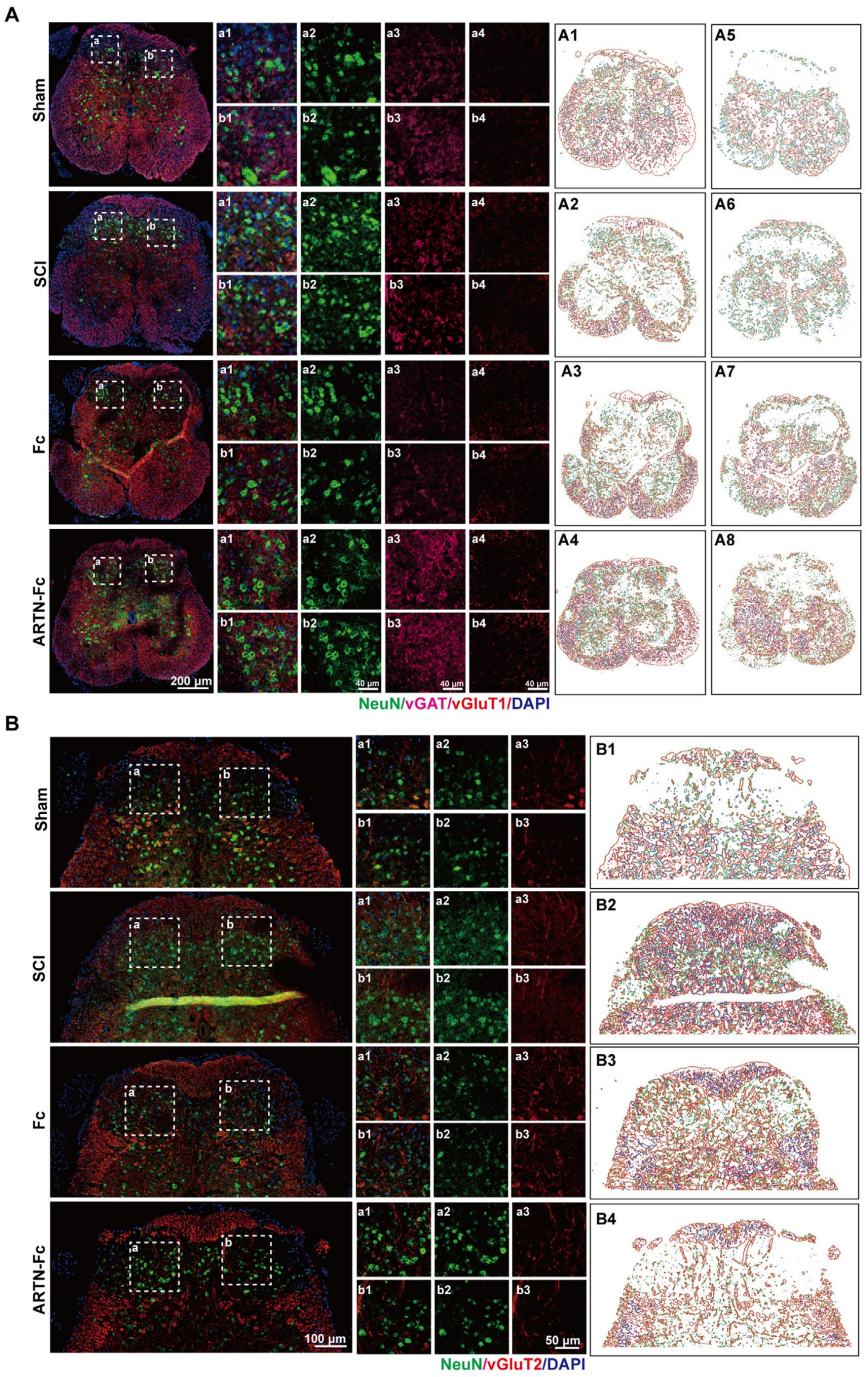
ARTN‐Fc modulates inhibitory/excitatory synaptic marker expression in the spinal dorsal horn after SCI. (A) Immunofluorescence staining of NeuN (green), vGAT (pink), vGluT1 (red) and DAPI (blue) in each group. Scale bar =200 μm. High magnification images showing the dorsal horn region of the spinal cord. a1 represents a magnified image of area a. b1 indicates a magnified image of region b. a2–4 represents the green, pink, and red single‐channel images of a1, respectively. b2–4 represents the green, pink, and red single‐channel images of b1, respectively. Scale bar = 40 μm. (B) Immunofluorescence staining of NeuN (green), vGluT2 (red) and DAPI (blue) in each group. Scale bar = 100 μm. High magnification images showing the dorsal horn region of the spinal cord. a1 represents a magnified image of area a. b1 indicates a magnified image of region b. a2–3 represents the green and red single‐channel images of a1, respectively. b2–3 represents the green and red single‐channel images of b1, respectively. Scale bar = 50 μm. A1–A4 represents the contour map of vGAT^+^ cells excluding DAPI interference, A5–A8 represents the contour map of vGluT1^+^ cells excluding DAPI interference, and B1–B4 represents the contour map of vGluT2^+^ cells excluding DAPI interference. The red line represents the contour with density 50. The green line represents the contour with density 35. The blue line represents the contour with density 30.

Immunofluorescence staining showed that vGAT‐positive inhibitory synaptic signals were reduced in the SCI and Fc groups compared with the Sham group, whereas ARTN‐Fc treatment increased vGAT expression. In contrast, no obvious difference in vGluT1‐positive excitatory synaptic density was observed among the groups (Figure 8A). To further support the changes in the inhibitory GABAergic system, we examined GAD1, a key enzyme involved in GABA synthesis. Immunofluorescence staining and Western blot analysis showed that GAD1 expression was reduced after SCI and partially restored after ARTN‐Fc treatment (Figure S4A–D). These results suggest that ARTN‐Fc treatment is associated with enhanced inhibitory GABAergic marker expression in the dorsal horn.

We further examined vGluT2, a vesicular glutamate transporter closely related to nociceptive transmission in the spinal dorsal horn. vGluT2 expression was increased in the SCI and Fc groups, while ARTN‐Fc treatment reduced vGluT2 expression in the dorsal horn region (Figure 8B). Taken together, ARTN‐Fc treatment increased inhibitory markers, including vGAT and GAD1, and reduced the excitatory marker vGluT2 without obvious changes in vGluT1. These findings suggest that ARTN‐Fc alleviates hyperalgesia in association with a shift in inhibitory/excitatory synaptic marker expression in the L4–L5 dorsal horn.

## 4 Discussion

The glial cell line‐derived neurotrophic factor‐related family ligands, which include GDNF, Persephin (PSPN), Neurturin (NRTN), and Artemin (ARTN), offer potential therapeutic benefits. ARTN, in particular, supports the survival of sensory neurons primarily through interactions with GFRα3 [37]. Our studies have demonstrated that intrathecal administration of an ARTN‐Fc fusion protein promotes the regrowth of nerve fibers and improves the restoration of motor abilities in cases of spinal cord injury. The TLR4/NF‐κB signaling pathway appears to be involved in the ARTN‐Fc‐mediated regulation of microglial polarization and the development of neuropathic pain. Additionally, our findings suggest that modulating the excitatory/inhibitory synaptic marker expression within the dorsal horn of the lumbar spinal cord may contribute to a reduction in thermal hyperalgesia and inflammatory pain.

Although Fc protein was included as a control, this design cannot fully distinguish the effects of the ARTN domain from those of the ARTN‐Fc fusion construct. In our experiments, Fc alone showed limited effects on selected pro‐inflammatory markers, suggesting that the observed benefits were unlikely to be solely attributable to the Fc fragment. Therefore, the present findings should be interpreted as effects of the ARTN‐Fc fusion protein rather than free ARTN alone. Future studies using free ARTN, Fc‐silent ARTN‐Fc variants, or Fc receptor‐blocking strategies are needed to clarify the respective contributions of ARTN‐ and Fc‐related effects. Another limitation is the lack of pharmacokinetic and CNS distribution data for ARTN‐Fc. Although 50 μg/kg was selected based on preliminary testing, its stability, retention, and distribution after intrathecal injection remain unclear.

Microglia coordinate the injury response of glia and infiltrating white blood cells in the central nervous system. Excessive consumption of microglia will aggravate tissue injury and lead to deterioration of function [10, 38]. In a model of ischemia‐hypoxic‐induced retinal injury, ARTN has been demonstrated to have a significant impact on the regulation of post‐ischemia‐hypoxia inflammation (P17) [39]. Consistently, our results showed that ARTN‐Fc reduced inflammatory activation and tissue damage at 28 days after SCI, as reflected by decreased pro‐inflammatory markers and enhanced anti‐inflammatory phenotype markers. These findings suggest that ARTN‐Fc may contribute to SCI repair by limiting sustained microglia/macrophage‐mediated inflammation.

Targeting TLR4/NF‐κB signaling has been shown to attenuate neuroinflammation and neuropathic pain in the central nervous system. Previous studies reported that pharmacological inhibition of TLR4 or NF‐κB reduced microglial activation and inflammatory cytokine production in nerve injury‐related pain models [40, 41]. Research findings have indicated that peripherally derived Artemin (ARTN) exerts a regulatory role in pathological conditions associated with inflammatory and neuropathic pain [20]. Inflammatory conditions lead to an upregulation of ARTN, which subsequently mediates signal transduction involved in injury response [21]. The results of this investigation offer novel proof indicating that ARTN may modulate inflammation to facilitate the repair of spinal cord injuries, potentially by inhibiting the TLR4/NF‐κB signaling pathway.

Artemin‐GFRα3 signaling selectively mediates cold allodynia, and blocking this pathway can relieve cold pain without affecting heat or mechanical pain sensitivity [42]. Inhibition of Artemin can mitigate receptor activation and sensitization, thereby reducing inflammation‐induced bone marrow damage [43]. In contrast, peripheral ARTN administration can induce transient thermal hyperalgesia or increase mechanical and thermal pain sensitivity by regulating TRPV1/TRPA1 expression in DRG neurons [20, 44]. These findings suggest that peripheral ARTN signaling may promote nociceptor sensitization under certain inflammatory conditions. However, ARTN‐related signaling has also been associated with analgesic effects in other contexts. For example, the ARTN/RET‐related small molecule BT13 reduces mechanical hypersensitivity and normalizes DRG sensory neuron function [45], clinical studies have supported the development of ARTN (BG00010) for lumbosacral radiculopathy‐associated pain [46], and restoration of spinal Artemin levels reduces neuropathic pain after peripheral nerve injury [24]. These seemingly contradictory findings may be explained by the context‐dependent role of ARTN signaling in pain regulation. Under peripheral inflammatory conditions, increased ARTN may directly sensitize nociceptive afferents in the DRG by regulating pain‐related channels such as TRPV1, TRPA1, or TRPM8, thereby promoting thermal, mechanical, or cold hypersensitivity. In contrast, in nerve injury or SCI models, appropriate restoration or intrathecal delivery of ARTN‐related signaling may act predominantly within the injured spinal cord microenvironment, where neurorepair and neuroimmune modulation become more relevant. In our study, ARTN‐Fc was administered intrathecally after SCI and was associated with reduced microglia/macrophage‐mediated neuroinflammation, suppressed TLR4/NF‐κB‐related signaling, and altered inhibitory/excitatory synaptic marker expression. Therefore, ARTN may exert either pro‐ or anti‐nociceptive effects depending on the disease model, route of administration, dose, timing, tissue distribution, and cellular targets.

Although the causes of neuropathic pain are diverse, altered excitatory and inhibitory signaling in the spinal dorsal horn is a common mechanism underlying central sensitization and hyperalgesia [47]. In the pain pathway, brain cells have the ability to secrete GABA and inhibit pain signal transduction [48]. In particular, loss of GABAergic inhibition and enhancement of glutamatergic excitation can increase dorsal horn neuronal excitability after SCI. GAD1, which encodes glutamate decarboxylase 67, is a key enzyme responsible for GABA synthesis and is commonly used as a marker of GABAergic inhibitory neurons. Reduced GAD1 expression may therefore reflect impaired inhibitory regulation in the dorsal horn [49, 50]. In contrast, vGluT2 is mainly expressed in the spinal cord, thalamus, and lower brainstem and is closely associated with nociceptive glutamatergic transmission. Previous studies have shown that reduced vGluT2 signaling can alleviate neuropathic pain symptoms [51]. It is noteworthy that significantly inhibiting nociceptive hypersensitivity induced by sciatic nerve injury in the dorsal horn of spinal cord vGluT2 neurons by knocking down Tiam1 in vGluT2 neurons [52]. vGluT1 and vGluT2 show complementary expression patterns and participate in sensory transmission [53]. In our SCI model, vGAT and GAD1 expression were decreased, whereas vGluT2 expression was increased in the L4–L5 spinal dorsal horn after injury. ARTN‐Fc treatment partially reversed these changes, as shown by increased inhibitory GABAergic markers and reduced vGluT2 expression, while vGluT1 expression remained largely unchanged. These findings suggest that ARTN‐Fc may alleviate hyperalgesia in association with altered inhibitory/excitatory synaptic marker expression in the spinal dorsal horn. However, these marker‐based results do not directly demonstrate functional synaptic transmission. Future studies using electrophysiological recordings as well as further investigation of dorsal horn and dorsal root ganglion glutamatergic/GABAergic signaling are needed to clarify how ARTN‐Fc modulates pain‐related synaptic activity after SCI.

## 5 Conclusions

This study demonstrates that ARTN‐Fc fusion protein enhances functional recovery and alleviates neuropathic pain after spinal cord injury (SCI). Mechanistically, ARTN‐Fc suppresses TLR4/NF‐κB‐associated neuroinflammation, attenuates NLRP3 inflammasome/pyroptosis‐related signaling, and modulates inhibitory/excitatory synaptic marker expression in the spinal dorsal horn.

## Author Contributions

Zhouguang Wang, Sipin Zhu, and Sunren Sheng conceived the study. Wenjie Lu, Yurong Tu, and Renkai Wang performed the experiments. Minghao Jiang provided behavioral analysis tools. Minghao Jiang, Junyu Zhuang, and Jiahui Song analyzed the data. Ping Wu offered suggestions. Wenjie Lu and Yurong Tu wrote the manuscript. All authors have read and approved the article.

## Funding

This study was partly funded by grants from the National Natural Science Funding of China (82372396, 82172424), Zhejiang Medical and Health Science and Technology Plan Project (2022RC210), National College Student Innovation and Entrepreneurship Training Program (202310343052), and Wenzhou Major Science and Technology Innovation Project Approval Project (ZY2022007).

## Ethics Statement

All animal‐related procedures received approval from the Animal Care and Use Committee of Wenzhou Medical University (No. wydw2024‐0029).

## Conflicts of Interest

The authors declare no conflicts of interest.

## Supporting information


**Figure S1:** ARTN‐Fc regulates inflammatory phenotype marker expression through NF‐κB signaling in LPS‐stimulated BV2 cells. BV2 cells were divided into the following groups: Control, LPS, LPS + Fc, LPS + ARTN‐Fc, LPS + BAY 11–7082, and LPS + ARTN‐Fc + TNF‐α. (A) Representative immunofluorescence images of iNOS (red) and Iba1 (green) in BV2 cells. Nuclei were counterstained with DAPI (blue). Scale bar = 50 μm. (B) Representative immunofluorescence images of Arg‐1 (red) and Iba1 (green) in BV2 cells. Nuclei were counterstained with DAPI (blue). Scale bar = 50 μm. (C, D) Quantitative analysis of iNOS and Arg‐1 mean fluorescence intensity, expressed as percentage of the Control group (*n* = 6). (E) Representative Western blot images showing the protein levels of iNOS and Arg‐1 in BV2 cells from each group. β‐actin was used as an endogenous control. (F, G) Quantitative analysis of relative iNOS and Arg‐1 protein expression (*n* = 3). Statistical analysis was performed using one‐way ANOVA followed by Tukey's multiple comparisons test. *p < 0.05, **p < 0.01, n.s. = not significant.
**Figure S2:** Preliminary dose‐exploration of ARTN‐Fc in SCI mice. Mice were divided into six groups: Sham, SCI, Fc, ARTN‐Fc 25 μg/kg, ARTN‐Fc 50 μg/kg, and ARTN‐Fc 100 μg/kg. (A) Representative Western blot images showing the protein expression of iNOS and Arg‐1 in spinal cord tissues. β‐actin was used as an endogenous control. (B, C) Quantitative analysis of relative iNOS and Arg‐1 protein levels (*n* = 3). (D) Representative Western blot images showing the protein expression of TLR4, p‐p65, and p65 in spinal cord tissues. β‐actin was used as an endogenous control. (E) Quantitative analysis of relative TLR4 protein expression (*n* = 3). (F) Quantitative analysis of the p‐p65/p65 ratio (*n* = 3). Statistical analysis was performed using one‐way ANOVA followed by Tukey's multiple comparisons test. *p < 0.05, **p < 0.01, n.s. = not significant.
**Figure S3:** ARTN‐Fc alleviates inflammatory pain and thermal pain after spinal cord injury. (A) Formalin was injected into the hind paw of mice. c‐fos was expressed by immunofluorescence in the lumbar spinal dorsal horn, scale bar =200 μm. A boxed region illustrates a representative region with high power images, scale bar = 40 μm. (B) Quantitative analysis of c‐fos positive cells in dorsal horn region (*n* = 6). (C) Quantitative analysis of hind paw withdrawal latency time in a 49°C water bath (*n* = 9). c‐Fos‐positive cells were analyzed by one‐way ANOVA followed by Tukey's multiple comparisons test. Paw withdrawal latency was analyzed by two‐way repeated‐measures ANOVA followed by Tukey's multiple comparisons test. *p < 0.05, **p < 0.01, n.s. = not significant.
**Figure S4:** ARTN‐Fc enhances GAD1 expression in the L4–L5 spinal dorsal horn after SCI. (A) Immunofluorescence staining of GAD1 (red) and DAPI (blue) in each group. Scale bar =100 μm. High magnification images showing the dorsal horn region of the spinal cord. Scale bar =30 μm. (B) Quantitative analysis of GAD1 mean fluorescence intensity in the L4–L5 spinal dorsal horn (*n* = 5). (C) Representative Western blot images showing GAD1 protein expression in L4–L5 spinal cord segments. β‐actin was used as an endogenous control. (D) Quantitative analysis of relative GAD1 protein levels (*n* = 3). Statistical analysis was performed using one‐way ANOVA followed by Tukey's multiple comparisons test. *p < 0.05, **p < 0.01, n.s. = not significant.

## Data Availability

The data supporting the results of this study can be provided upon the request of the corresponding author.
