# Supplementary material for: Recombinant Artemin‐Fc Fusion Protein Attenuates TLR4/NF‐κB‐Associated Neuroinflammation and Modulates Inhibitory/Excitatory Synaptic Marker Expression After Spinal Cord Injury
Source: CNS Neurosci Ther. 2026 Aug 13;32(8):e71086. doi: 10.1002/cns.71086 (PMC13473840; doi:10.1002/cns.71086)
Supplement: Supplementary file 1 [file CNS-32-e71086-s001.doc]

**Fig. S1. ARTN-Fc regulates inflammatory phenotype marker expression through NF-κB signaling in LPS-stimulated BV2 cells.** BV2 cells were divided into the following groups: Control, LPS, LPS + Fc, LPS + ARTN-Fc, LPS + BAY 11-7082, and LPS + ARTN-Fc + TNF-α. (A) Representative immunofluorescence images of iNOS (red) and Iba1 (green) in BV2 cells. Nuclei were counterstained with DAPI (blue). Scale bar = 50 μm. (B) Representative immunofluorescence images of Arg-1 (red) and Iba1 (green) in BV2 cells. Nuclei were counterstained with DAPI (blue). Scale bar = 50 μm. (C–D) Quantitative analysis of iNOS and Arg-1 mean fluorescence intensity, expressed as percentage of the Control group (n = 6). (E) Representative Western blot images showing the protein levels of iNOS and Arg-1 in BV2 cells from each group. β-actin was used as an endogenous control. (F–G) Quantitative analysis of relative iNOS and Arg-1 protein expression (n = 3). Statistical analysis was performed using one-way ANOVA followed by Tukey’s multiple comparisons test. *p < 0.05, **p < 0.01, n.s. = not significant.

**Fig. S2. Preliminary dose-exploration of ARTN-Fc in SCI mice.** Mice were divided into six groups: Sham, SCI, Fc, ARTN-Fc 25 μg/kg, ARTN-Fc 50 μg/kg, and ARTN-Fc 100 μg/kg. (A) Representative Western blot images showing the protein expression of iNOS and Arg-1 in spinal cord tissues. β-actin was used as an endogenous control. (B–C) Quantitative analysis of relative iNOS and Arg-1 protein levels (n = 3). (D) Representative Western blot images showing the protein expression of TLR4, p-p65, and p65 in spinal cord tissues. β-actin was used as an endogenous control. (E) Quantitative analysis of relative TLR4 protein expression (n = 3). (F) Quantitative analysis of the p-p65/p65 ratio (n = 3). Statistical analysis was performed using one-way ANOVA followed by Tukey’s multiple comparisons test. *p < 0.05, **p < 0.01, n.s. = not significant.

**Fig. S3. ARTN-Fc alleviates inflammatory pain and thermal pain after spinal cord injury.** (A) Formalin was injected into the hind paw of mice. c-fos was expressed by immunofluorescence in the lumbar spinal dorsal horn, scale bar =200 μm. A boxed region illustrates a representative region with high power images, scale bar = 40 μm. (B) Quantitative analysis of c-fos positive cells in dorsal horn region (n = 6). (C) Quantitative analysis of hind paw withdrawal latency time in a 49℃ water bath (n = 9). c-Fos-positive cells were analyzed by one-way ANOVA followed by Tukey’s multiple comparisons test. Paw withdrawal latency was analyzed by two-way repeated-measures ANOVA followed by Tukey’s multiple comparisons test. *p < 0.05, **p < 0.01, n.s. = not significant.

**Fig. S4. ARTN-Fc enhances GAD1 expression in the L4–L5 spinal dorsal horn after SCI.** (A) Immunofluorescence staining of GAD1 (red) and DAPI (blue) in each group. Scale bar =100 μm. High magnification images showing the dorsal horn region of the spinal cord. Scale bar =30 μm. (B) Quantitative analysis of GAD1 mean fluorescence intensity in the L4–L5 spinal dorsal horn (n = 5). (C) Representative Western blot images showing GAD1 protein expression in L4–L5 spinal cord segments. β-actin was used as an endogenous control. (D) Quantitative analysis of relative GAD1 protein levels (n = 3). Statistical analysis was performed using one-way ANOVA followed by Tukey’s multiple comparisons test. *p < 0.05, **p < 0.01, n.s. = not significant.
